# Immunopathogenesis of polymicrobial otitis media

**DOI:** 10.1189/jlb.0709518

**Published:** 2009-10-20

**Authors:** Lauren O. Bakaletz

**Affiliations:** ^1^ The Research Institute at Nationwide Children's Hospital, Center for Microbial Pathogenesis, The Ohio State University College of Medicine, Columbus, Ohio, USA

**Keywords:** ear infection, innate immunity, nontypeable *Haemophilus influenzae*, *Streptococcus pneumoniae*, *Moraxella catarrhalis*, Eustachian tube

## Abstract

OM, or inflammation of the middle ear, is a highly prevalent infection in children worldwide. OM is a multifactorial disease with multiple risk factors, including preceding or concurrent viral URT infection. Hence, OM is also a polymicrobial disease. The mechanisms by which viruses predispose to bacterial OM are replete; however, all are predicated on the general principle of compromise of primary host airway defenses. Thus, despite an as‐yet incomplete understanding of the molecular mechanisms involved in bacterial superinfection of a virus‐compromised respiratory tract, the URT viruses are known to induce histopathology of airway mucosal epithelium, up‐regulate expression of eukaryotic receptors used for bacterial adherence, alter the biochemical and rheological properties of airway mucus, and affect innate and acquired host immune functions, among others. Although discussed here in the context of OM, during preceding or concurrent viral infection of the human respiratory tract, viral impairment of airway defenses and the resulting predisposition to subsequent bacterial coinfection are also known to be operational in the mid and lower airway as well.

## Abstract 2

The synergistic relationship between URT viruses and bacteria in OM pathogenesis is not fully understood, but overall is predicated on viral impairment of airway defenses.

**Abbreviations**
AOM: acute otitis media
AP: antimicrobial peptide
AV: adenovirus
cBD‐1: chinchilla β‐defensin‐1
IN: intranasal
LTB4: leukotriene B4
MEF: middle ear fluid
NP: nasopharynx
NTHI: nontypeable *Haemophilus influenzae*
OM: otitis media
OME: otitis media with effusion
PMN: polymorphonuclear neutrophil
RSV: respiratory syncytial virus
URT: upper respiratory tract

## MULTIFACTORIAL AND POLYMICROBIAL ETIOLOGY OF OM

OM is a term used broadly to describe AOM as well as OME and refers to an inflammation of the middle ear cavity or tympanum (http://www.expert-reviews.com/doi/abs/10.1586/erv.09.63), regardless of etiology. AOM is defined as the presence of MEF (or effusion) in conjunction with rapid onset of symptoms that can include otalgia, otorrhea, fever, and irritability [1]. OME is defined as the presence of fluid in the middle ear in the absence of signs or symptoms of an ear infection [1]. OM can be a purely viral infection; however, in childhood, an “ear infection” is more typically considered to be the bacterial component of a viral‐bacterial coinfection. OM is highly prevalent worldwide, resulting in extensive morbidity and socioeconomic burden. In 1990, there were 24.5 million physicians’ office visits made for OM in the United States, representing a greater than 200% increase compared with that reported in the 1980s [2]. Worldwide, it is reported that between 65 and 330 million children suffer from chronic secretory OM, 60% of which have an associated hearing loss [3, 4, 5]. As such, OM is the most frequently diagnosed illness in children under 15 years of age and is the primary cause for Emergency Room visits [6]. Shockingly, in 1990, ∼28,000 childhood deaths were attributed to OM worldwide [5]. Although today, OM is only rarely associated with mortality in developed countries, the associated morbidity is nonetheless significant. The most common sequelae of OM [7, 8] is hearing loss with behavioral, educational, and language development delays as additional consequences of early‐onset OM [9, 10]. Socioeconomically, total costs of diagnosing and managing OM exceed $5 billion annually in the United States alone [11, 12, 13, 14].

Despite its prevalence, classically virulent microbes do not cause OM. Instead, it is caused by members of the normal flora of the pediatric NP, which are capable of causing disease when they gain access to more privileged sites within the upper or lower reaches of the airway [15]. The three predominant bacterial pathogens of OM are *Streptococcus pneumonia*e, NTHI, and *Moraxella catarrhalis* [16], and whereas of these three microbes, *S. pneumoniae* is more likely to be capable of behaving in a virulent manner (i.e., when inducing such diseases as invasive pneumococcal disease or pneumonia), in the disease course of OM, when residing as normal flora members, none of these microbes induces inflammation or any sign of infection. It is only under compromising conditions that these microbes ascend the Eustachian tube from their colonizing site in the NP, gain access to the middle ear, and induce disease.

Multiple conditions can predispose to the development of disease by these normal flora members; hence, OM is considered to be multifactorial. Risk factors for OM include male gender, familial predisposition, environment, attendance at daycare, bottle‐feeding, use of pacifier, immunological immaturity, anatomy, and previous or concurrent viral infection [17], among others. That viral URT infection predisposes to bacterial OM defines OM as not only multifactorial but also as polymicrobial (Table 1). The crucial role of the URT viruses in the pathogenesis of bacterial OM is well established and has been the subject of several excellent reviews [62, 63, 64, 65, 66, 67, 68, 69].

**Table 1:** General Mechanisms of URT Virus Predisposition to Bacterial OM

Influence expression of receptors used by bacteria for adherence [18, 19, 20, 21, 22, 23, 24, 25, 26, 27, 28, 29, 30, 31]
Alter biochemical and rheological properties of airway mucus [19, 31, 32, 33, 34, 35, 36, 37]
Induce histopathology of airway epithelium with compromise of Eustachian tube functions [38, 39, 40, 41, 42, 43]
Alter the efficacy of antibiotics [44, 45, 46, 47, 48, 49, 50]
Effect host immune function (innate and acquired), including induction of inflammatory mediators [51, 52, 53, 54, 55, 56, 57, 58, 59, 60, 61]

## MECHANISMS OF VIRAL PREDISPOSITION TO BACTERIAL OM

Whereas there are still gaps in our understanding, multiple mechanisms have been identified that serve as contributing factors in the synergistic relationship between the URT viruses and the primary bacterial pathogens of OM, and all fall within the general category of compromise of airway defenses (Table 2). In most cases, it is highly likely that more than one of these mechanisms is operational at any given time and quite possibly varies depending on time‐point within the multifactorial disease course [80, 81]. Greater detail about several of these specific mechanisms is offered below.

**Table 2:** Microorganisms Most Commonly Associated with OM

Viruses [16, 70, 71]	Bacteria [16]
Rhinovirus	*S. pneumoniae*
RSV	NTHI
Parainfluenza virus	*M. catarrhalis*
Influenza A & B viruses	Group A streptococci
AV	
Coronavirus	
Enterovirus	
Emerging microorganisms associated with OM	
Human bocavirus [72, 73]	*Alloiococcus otitidis* (status as an OM pathogen is debated) [74, 75, 76, 77]
Human metapneumovirus [78]	
Human rhino‐enterovirus [72, 79]	

### Viral effects on bacterial adherence and/or colonization

One mechanism for the commonly observed association between a concurrent URT virus infection and subsequent bacterial superinfection is a result of the fact that cells infected with certain viruses are more permissive to bacterial adherence, ultimately leading to secondary infection and disease [18]. The relevance of this hypothesis to pathogenesis of OM has been demonstrated as a result of the fact that many URT viruses do indeed augment adherence by specific bacterial pathogens of OM. For example, influenza A virus increases the adherence of *S. pneumoniae* to mouse tracheal epithelial cells [19] but not of NTHI to chinchilla tracheal epithelium in organ culture [20]. Infection with AV types 1, 2, 3, and 5 significantly enhances the binding of adherent strains of *S. pneumoniae*, which had been isolated from the NP of children with frequent episodes of AOM, to human lung epithelial cells in vitro [21]. Similarly, RSV infection of A549 cells significantly enhances attachment by NTHI that specifically express one of several known adhesins [22]. By flow cytometry, El Ahmer et al. [23] showed that viral infection significantly increased adherence by all three groups of microorganisms most commonly associated with AOM and chronic OM to influenza A virus‐infected Hep‐2 cells. Through the use of a panel of mAb directed against specific cell‐surface antigens, these latter investigators found that infection of Hep‐2 cells with influenza A virus resulted in a significant increase in expression of known receptors for adherence used by several Gram‐negative bacteria. More recently, it was shown that URT viruses can induce up‐regulated expression of carcinoembryonic antigen‐related cell adhesion molecule 1, ICAM1, and platelet‐activating receptor, additional eukaryotic receptors known to be used for adherence by multiple human mucosal pathogens [24, 25, 26]. Thus, virus‐induced up‐regulation of host cell‐surface antigens that serve as bacterial receptor sites appears to be a common theme in the pathogenesis of OM as well as other diseases of the respiratory tract.

As bacterial invaders of the middle ear are those microorganisms that normally, benignly colonize the NP, gaining the understanding that viral infection could promote bacterial adherence to the airway epithelial cell in vitro implied a key molecular mechanism that underlied the disease course of OM. In man, more type I pneumococci and *Haemophilus influenzae* adhere to pharyngeal cells collected from human volunteers experimentally infected with influenza A/USSR/77 than to cells collected prior to challenge [27]. Exposure of cotton rats to RSV increased colonization of respiratory tract epithelium by NTHI [28]. In the chinchilla, influenza A virus (but not AV) similarly enhances NP colonization by *S. pneumoniae*, particularly by a strain with an opaque versus a transparent phenotype [29, 30]. Colonization of mice by NTHI and *S. pneumoniae* increased after influenza A virus infection [31].

Studies in human challenge models, wherein influenza A virus infection was shown to promote colonization with *S. pneumoniae*, have corroborated the positive correlation between viral URT infection and NP colonization [82]. Increased and/or early NP colonization of children by bacteria associated with OM has been positively correlated with an increased incidence of OM [83, 84]. Even in the absence of a concurrent viral URT infection, children are colonized with the organisms that induce OM soon after birth. At 6 months of age, 26% of infants are colonized already with *M. catarrhalis*, 24% with *S. pneumoniae*, and 9% with NTHI [85]. By 1 year of age, these percentages increased to 72%, 54%, and 33%, respectively. Early colonization is associated with early, initial episodes of AOM, and colonization with *S. pneumoniae* or *H. influenzae* in the first year of life increases the risk of becoming otitis‐prone fourfold. Up to 10% of children are considered otitis‐prone, which is defined as having four or more episodes of AOM within 1 year or having experienced at least 8 months during which there is an effusion present within the middle ear space [85]. There is an additional, direct relationship between how frequently children are colonized by the pathogens of OM and the frequency of occurrence of AOM [85]. Thus, infections of the middle ear and particularly, those that occur early in life can induce pathological changes in the middle ear that set the stage for subsequent recurrent or chronic OM. Prevention of these initial episodes of OM, regardless of etiology, is thereby a priority consideration of vaccine development efforts.

### Viral compromise of Eustachian tube function

Virus‐induced damage to the mucosa that lines the uppermost airway and the effect that this pathology has on airway function, particularly that of the Eustachian tube, contribute significantly to bacterial invasion of the middle ear [38, 39]. In children, the Eustachian tube is shorter and more horizontal in orientation than in adults. The immature Eustachian tube is also more compliant (or “floppy”), as engagement of the *tensor veli palatini* muscle (which holds mature Eustachian tubes closed passively) is not yet complete. These attributes make the pediatric Eustachian tube naturally more susceptible to retrograde ascent by microbes that typically colonize the NP; however, this susceptibility is enhanced significantly during times of URT viral compromise.

The effect of influenza A virus on chinchilla Eustachian tube function after IN challenge was investigated by Giebink et al. [40] in an attempt to better understand the physiological mechanisms responsible for the characteristic negative pressure recorded in the middle ears of animals infected with any of several strains of this virus. Virus‐induced inflammation of the tympanic membrane and the under‐pressured state of the middle ear mirrored histopathological damage to Eustachian tube epithelium as well as the accumulation of mucus and cellular debris present within the tubal lumen. Epithelial damage was greatest in the proximal two‐thirds of the Eustachian tube, whereas goblet cell metaplasia and increased secretory activity predominated in the distal or “tympanic” one‐third of this tubal organ. These data thus provided the first morphologic correlate for the development of an underpressured middle‐ear state and contributed significantly to our understanding of the basis for bacterial OM during viral URT infection.

Overall, the findings in animal models correlate well with studies of human volunteers challenged with rhinovirus or influenza A virus. Subsequent to IN challenge with rhinovirus, volunteers that developed a “cold” [41, 42] demonstrated abnormal middle‐ear pressures, decreased nasal patency, and depressed tubal function in 50% or more of ears. In another study, more than 80% of volunteers inoculated with influenza A virus developed Eustachian tube dysfunction and middle‐ear underpressures 4 and 5 days after challenge [43]. Five of 21 subjects with low prechallenge antibody titers to influenza A virus also developed OM. Collectively, these data support a causal relationship among viral URT infection, Eustachian tube obstruction, abnormal under‐pressured middle‐ear state, and development of bacterial OM.

The degree to which a particular virus compromises the airway and particularly, the protective functions of the Eustachian tube has a tremendous influence on whether bacterial OM will develop and how severe the disease course will be. Giebink and Wright [86] clearly showed this correlation when they reported that strains of influenza A virus had markedly different levels of virulence in the chinchilla host when development of a negative middle‐ear pressure, neutrophil dysfunction, and increased susceptibility to pneumococcal OM included the outcomes measured. These data thus supported epidemiological evidence that had already shown there to be striking differences in AOM prevalence in association with different influenza A outbreaks.

As a general principle, it is thus believed that although nearly all URT viruses and some enteroviruses can predispose to bacterial OM, there is intra‐ and interstrain variability in their relative ability to do so. Among the viruses, influenza virus, parainfluenza virus, rhinovirus, coronavirus, RSV, and AV are those most commonly linked with AOM [70, 87, 88]; however, of these, rhinovirus and RSV are often more commonly identified than others [89]. Whereas rhinoviruses are typically more frequently identified by culture and other molecular mechanisms, RSV is more commonly associated with concurrent AOM. This strong association between RSV and concurrent AOM has been reported by many laboratories [66, 68, 90, 91, 92, 93, 94, 95, 96]. Overall, viral URT infections that induce more severe compromise of the uppermost airway in terms of its physiological and/or defensive immune functions are typically associated with the most enhanced ability to predispose to bacterial OM.

In animal models, the period of time between initial viral infection and bacterial invasion of the middle ear is ∼9 days for *S. pneumoniae* after influenza A inoculation [97] and 7–10 days for NTHI following AV inoculation [98]. This period of time is similar to the interval between onset of symptoms of URT infection and AOM in children (http://www.journals.uchicago.edu/doi/abs/10.1086/528685). In a study of 204 cases of AOM that occurred in association with viral URT infection, 75% of cases occurred within 1 week of onset of symptoms, and 85% occurred within the first 9 days [99]. In an examination of 250 episodes of AOM in children, Koivunen et al. [100] similarly found that 63% of AOM occurred in the 1st week of URT virus infection, and 89% occurred by the end of the 2nd week after onset of URT infection. The greatest incidence of AOM was observed 2–5 days after the onset of respiratory symptoms. Collectively, these observations fit well with the physician and parent‐described phenomenon that “…*my child gets a cold, then a week later has an ear infection*” [62].

### Viral effects on antibiotic efficacy

Arola et al. [44] and Chonmaitree et al. [45] first suggested the association between viral URT infection and antibiotic treatment failure in children with OM. Although treatment guidelines have since been revised, at one time, antibiotic therapy was recommended for all patients with AOM. In 4–18% of these patients, however, signs of infection persisted, despite the fact that in ∼81% of these cases, the microbes isolated from the middle ears were susceptible to the prescribed antimicrobial [44]. Patients unresponsive to antibiotic treatment also tended to have positive viral cultures of the middle ear. In a later prospective study of 271 infants and children with AOM, there was evidence of viral infection in 46% of these patients, 76% of whom also had bacteria present in their MEFs [46]. Fifty‐one percent of the patients with a combined bacterial‐viral infection had persistent OM 3–12 days after institution of antibiotic therapy versus those with simple bacterial OM (35%) or those patients with purely viral OM (19%). Additional prospective studies have since corroborated the linkage between viral involvement in OM and antibiotic treatment failure [47, 48, 49]. Collectively, these data suggested that viral infection of the middle ear likely interferes directly or indirectly with the clinical response to antibiotic treatment.

One explanation for the commonality of treatment failure in cases of viral‐bacterial OM may lie in the combined effect of viral and bacterial invasion of the middle ear on penetration of antimicrobials from the bloodstream into the middle‐ear cavity. A prospective study of 34 children with AOM [50] evaluated whether viral coinfection reduced the efficacy of antibiotics by determining the penetration and pharmacokinetics of amoxicillin during bacterial or combined bacterial and viral AOM. Toward this end, geometric mean amoxicillin concentrations were determined using MEFs, nasal wash fluids, and serum collected from all 34 children at selected times between 0.5 and 4 h after oral dosing (40 mg/kg/day). Virus‐only‐infected children had the lowest values (2.7 μg/ml in MEFs), which were similar to those obtained in children that were culture‐negative (2.9 μg/ml in MEFs). Geometric mean amoxicillin concentrations were greater in children with a combined viral‐plus‐bacterial infection (4.1 μg/ml in MEFs) but were greatest in those with bacterial‐only infections of the middle ear (5.7 μg/ml in MEFs). Thus, there was a lesser penetration of amoxicillin from serum into MEFs when virus was also present in the middle ear.

A potential link among treatment failure, viral infection of the middle ear, and levels of specific inflammatory mediators has also been investigated. Chonmaitree et al. [51] measured levels of IL‐8, a PMN chemotactic cytokine, and LTB4, a potent inflammatory product of PMNs, in 271 MEFs collected from 196 children with AOM (42% of whom had evidence of a viral URT infection as well). LTB4 and IL‐8 levels were significantly greater in children with bacterial AOM or mixed bacterial‐viral infection at the time of diagnosis compared with those children who had culture‐negative MEFs. When bacteria were absent, no virus‐related effect was observed for IL‐8; however, these levels were significantly greater in MEFs that contained bacteria and viruses than in all other groups. Moreover, in children that had bacteria and virus isolated from one MEF but were virus‐negative in the contralateral MEF, levels of IL‐8 and LTB4 were greater in the MEFs that contained bacteria and virus in all but one case. Treatment failure that occurred after 2–5 days of compliance with either of two antibiotic regimens was significantly associated with greater LTB4 levels in initial MEFs, whereas recurrence of AOM within 1 month was associated with increased IL‐8 levels in these effusions. Thereby, both of the studied PMN‐related inflammatory substances likely played a role in delayed recovery and/or recurrence of disease.

### Viral effect on host immune functions

The effect of URT viruses on host immune function is another topic of enormous interest as it relates to the pathogenesis of OM. The effect that viral URT infection has on neutrophil [52] and alveolar macrophage [53] function has been appreciated for some time, and transient peripheral PMN dysfunction has been reported in children with recurrent OM [54]. Further, PMN dysfunction has been linked with experimental influenza A virus infection in a chinchilla model [55]. Abramson et al. [55] reported significantly depressed chemotactic, chemiluminescent, and bactericidal activities by PMNs 4–8 days after viral challenge when compared with controls. The authors of this work hypothesized that the known greater susceptibility of chinchillas to development of pneumococcal OM after coinoculation with influenza A virus [97] might be, in part, a result of the demonstrated, impaired PMN chemotactic and oxidative microbiocidal activities.

Viral‐mediated release of cytokines and inflammatory mediators in the respiratory tract is also believed to contribute to the pathogenesis of OM. Cytokine activity in NP secretions collected during RSV infection of infants and children demonstrated local production of IL‐6 and TNF‐α in 100% and 67%, respectively [56, 57]. Similarly, experimental infection of 17 adult volunteers with influenza A virus induced significantly increased levels of IL‐6, but not IL‐4, in nasal lavage fluids of all 12 subjects who shed virus [58]. In five subjects who did not shed virus, increased levels of this pleotropic cytokine were not found. Others and Noah et al. [56, 57] examined children in a day care setting during acute URT viral infection, including children with OM, and found markedly elevated levels of IL‐1β, IL‐6, IL‐8, and TNF‐α in nasal lavage fluids.

The enhanced production of inflammatory mediators during a combined bacterial‐viral infection has also been shown to occur in the middle ear [59]. Histamine levels were measured in 677 MEF samples collected from 248 children with AOM, of which 47% had a documented viral infection as well. Histamine content was significantly greater in bacteria‐positive or virus‐positive fluids versus culture‐negative samples. Together, the presence of bacteria and viruses had an additive effect on histamine content in MEFs. Histamine is known to cause impaired ciliary activity and induce mucosal swelling in the tubotympanum, thus delaying mucociliary‐mediated clearance of fluid and/or debris from the tympanic cavity. High levels of histamine, cytokines, and other inflammatory mediators in MEFs can also lead to increased inflammation in the middle ear, which is, in essence, a double‐edged sword. Although an inflammatory response is necessary for microbial clearance, an overzealous host response to the presence of microbes (particularly endotoxin‐expressing, Gram‐negative bacteria) is believed to contribute to the pathogenesis and prolongation of OM via the associated tissue destruction and interference with penetration of antimicrobials into the site, among other mechanisms [67].

In the case of a multifactorial and polymicrobial disease such as OM, it is probably wisest to consider the relative potential benefit versus deficit of inflammatory changes in the middle ear to the host in terms of when they are occurring within the disease course and to what degree. Moreover, as the induced inflammation is highly variable and dependent on which microbes are working synergistically to cause the disease, in addition to the age, immune status, previous colonization by specific microbes, and prior OM history of the child (among many others), whether the balance is tipped more in favor of the microbe or more in favor of the host is similarly, highly variable. This uncertainty has led to an active field of investigation, wherein several laboratories are beginning to look at multiple aspects of the kinetics and dynamics of this inflammatory process in greater detail. A full discussion is beyond the scope of this review; however, a few examples include studies, wherein it has been observed that NTHI, the most common pathogen of OME, as well as of chronic and recurrent OM [101], can induce necrosis of PMNs, one of the first cellular defenders of the middle ear [102]. Whereas this could be seen as a deficit to the host, PMNs have been shown recently to release their dsDNA in the form of “nets” that can trap and kill associated bacteria [103]. Moreover, Miles and colleagues [104] have shown that dying and necrotic neutrophils are actually anti‐inflammatory, subsequent to release of α‐defensins. Conversely, Ed Swords and his colleagues [105, 106] have shown that when one of the bacterial pathogens of OM induces and then resides within a biofilm community in the middle ear or lung, it modifies itself so that the endotoxin expressed on the bacterial surface is less likely to induce inflammation, thereby reducing the likelihood that it will be cleared and ensuring longer term residence within its human host. Clearly, much remains to be elucidated with regard to the complex factors involved in induction and ultimately, resolution of OM.

### Viral effect on rheological properties of mucus and mucociliary transport

Many URT viruses, including those commonly associated with OM, induce ultrastructural abnormalities or selective destruction of ciliated cells in respiratory tract mucosal epithelium [19, 32, 33]. Both effects can compromise mucociliary clearance mechanisms throughout the respiratory tract, including in the nasal cavity of children with acute viral infection [33]. Park et al. [34] studied influenza A‐induced histopathology in chinchillas, including suppression of ciliary activity of the mucosa that lines the Eustachian tube, and found a delayed ability of this organ to move fluid out of the middle ear. Concurrent with the described virus‐induced morphological changes was compromise of chinchilla Eustachian tube functions with the maximum effects observed 7–14 days after viral infection [34]. Slowing of ciliary beating, combined with the discoordinated activity among ciliated cells that also occurs as a result of viral infection, leads to compromised Eustachian tube clearance function. Thus, the ability of the mucociliary escalator of the Eustachian tube to transport fluid out of the middle‐ear space was similarly diminished maximally on Days 7–14 after transbullar challenge. Influenza A virus infection increased transport time from a normal value of ∼2.5 min to a situation in which dye transport by the Eustachian tube could not be demonstrated, even 15 min after its instillation into the middle ear. A similar decreased ciliary activity and increased time to clearance were reported in guinea pigs inoculated intratympanically with influenza A virus [35].

AV will also compromise Eustachian tube function in chinchillas [36]. However, for this URT virus, maximal compromise occurs 10–21 days after IN or 7–14 days after transbullar inoculation. In addition to focal necrosis and sloughing of epithelium to reveal the basal cell layer, subepithelial hemorrhage and edema, intense PMN infiltration, clumping and shortening, or loss of cilia were observed in sections of the Eustachian tube recovered from these animals. The presence of intranuclear viral inclusions and marked hyperplasia of goblet cells were additional histopathological changes consistent with AV infection.

Despite the fact that the exact nature of the induced histopathology in the Eustachian tube and middle ear is virus‐specific, the net effect in all cases of URT viral infection is severe compromise of the protective functions of the Eustachian tube. This compromise results in a temporal loss of ability of this organ to function as a primary defense organ of the middle ear. At the point of maximal viral compromise of the epithelium lining the Eustachian tube, the ascension of the Eustachian tube by bacteria colonizing the NP can be demonstrated [37]. Return to homeostatic conditions, including normal epithelial organization, ciliary morphology, and mucociliary clearance function, can take 2–10 weeks in children [33]. Thus, the middle ear is most susceptible to ascending bacterial infection during these periods of recovery.

In animal models and humans, there appears to be some specificity of the inter‐relationship between the viral and bacterial pathogens. One explanation offered to explain the specificity of this synergy resides in the fact that several URT viruses are known to have a distinct effect on the character of the mucus secreted by epithelial cells that line the mammalian airway. For example, AV infection can lead to an increase in relative amount and viscosity of NP secretions in chinchillas challenged with a serotype 1 isolate [36]; however, there is no evidence that the biochemistry of these secretions has been altered [37]. The marked goblet cell hyperplasia noted to occur in the mucosa that lines the Eustachian tube during AV infection in chinchillas provides a possible mechanism for the hypersecretion of mucus observed in these animals. This hypersecretion, combined with a likely inability to adequately hydrate this increased amount of “normal” mucus, is postulated to account for the changes in the rheological properties of airway secretions. Importantly, this latter situation appears to favor ascension of the Eustachian tube and initiation of OM as a result of NTHI, which is known to adhere to mucus within the Eustachian tube lumen, thus facilitating its ascent into the middle ear from the NP.

Conversely, influenza A virus infection, which also leads to an increased production of respiratory secretions in the 1st week after exposure of human volunteers [107], does indeed alter the character of these respiratory fluids. Unlike the situation with AV, these secretions are not more viscous than normal, and there is a concomitant change in the biochemical character of the mucus blanket and epithelial cell‐surface carbohydrates. These changes have been attributed to the action of viral neuraminidase. In support of this assertion, Hirano et al. [31] examined the NP mucosa of mice inoculated with influenza A virus for changes in labeling patterns using a battery of lectins. Staining of the mucus blanket and epithelial cell surface with lectins specific for N‐acetylglucosamine or galactose residues (i.e., peanut agglutinin, succincyl‐wheat germ agglutinin, and *Bandeiraea Simplicifolia* lectin II) was increased significantly compared with controls. As terminal glycosylation sequences of epithelial cell‐surface and mucus carbohydrates can mediate adherence by microorganisms, the effects of viral infection on these glycoproteins have important implications for the pathogenesis of OM.

## MECHANISMS OF RSV‐MEDIATED PREDISPOSITION TO BACTERIAL OM

As mentioned above, RSV is the virus most commonly associated with concurrent AOM. Yet, despite its recognized role as a significant viral copathogen of this highly prevalent pediatric disease, the mechanisms by which RSV predisposes to bacterial invasion of the middle ear are not fully understood currently. In an attempt to better characterize these molecular mechanisms, a murine and chinchilla model of RSV infection of the uppermost airway was developed recently [108]. In these studies, using BALB/c mice, we showed that the respiratory and olfactory mucosae of the nasal cavity and the ciliated epithelium of the Eustachian tube could be infected by RSV; however, only a small number of infected cells could be detected, and there was limited pathology. Interestingly, we found that the chinchilla was more susceptible to RSV infection. Chinchilla‐infected IN with RSV manifested signs of URT that were dose‐dependent, and compromise of chinchilla Eustachian tube function was clearly demonstrable. Moreover, we observed goblet cell hyperplasia within the Eustachian tube mucosa and increased mucus secretion into the Eustachian tube lumen, which could provide additional means to predispose to bacterial superinfection of the tympanum.

### RSV effect on expression of effectors of innate immunity

In children, a significant increase in the NP bacterial load is positively correlated with development of OM [83, 84], and OM‐prone children are more heavily colonized compared with their nonotitis‐prone counterparts [109, 110]. These observations suggest that maintenance of a relatively low concentration of bacteria in the NP is likely important for protection of the middle ear. One mechanism to maintain a minimal bacterial load in the NP is through the action of APs, which are key components of the innate immune response that serve to limit bacterial colonization of mucosal surfaces [111, 112, 113, 114, 115]. APs that kill causative agents of OM at micromolar concentrations are operative in the tubotympanum [60, 116, 117, 118, 119].

As altered expression of even a single AP can impact the ability of bacteria to colonize a mammalian host [120, 121, 122], we hypothesized that virus‐mediated, dysregulated expression of effector(s) of innate immunity might promote increased bacterial colonization of the upper airway and thus, the subsequent development of OM. Toward this end, we showed recently that RSV infection diminishes cBD‐1 (the ortholog of human β‐defensin 3) mRNA and protein abundance in the URT of the chinchilla [60, 61]. To demonstrate the net collateral effect that RSV‐induced, altered expression of this defensin had on the bacterial load present in the URT, we used a polymicrobial infection model to demonstrate that RSV challenge prior to inoculation with NTHI resulted in a consistent increase in the concentration of NTHI, recoverable in nasopharyngeal lavage fluids, compared with a mock‐challenged cohort. In addition, we demonstrated that this increased load of NTHI within the chinchilla URT was maintained for at least 5 days, a time‐frame in which we also showed that the amount of cBD‐1 mRNA and protein within URT mucosa was reduced. In further support of our hypothesis that virus‐mediated, altered expression of APs contributes to dysregulation of the load of NTHI residing within the NP, we also showed that delivery of anti‐(r)cBD‐1 to the chinchilla nasal cavity effectively *increased* the relative load of NTHI recovered from NL fluids by tenfold, compared with animals that received preimmune serum. We also provided evidence that IN delivery of exogenous (r)cBD‐1 to the chinchilla URT *decreased* the concentration of NTHI recovered by NL and the number of bacteria that were adherent to homogenized airway mucosae. These data provided the first evidence that virus‐induced dysregulation of AP expression contributes to the increased bacterial colonization known to precede development of OM.

**Figure 1:**
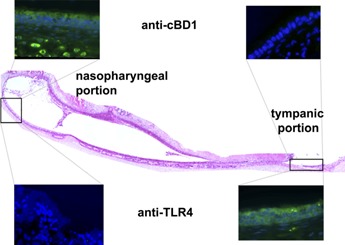
**Relative spatial distribution of expression of an innate immune effector (cBD‐1) versus that of TLR‐4 by the mucosa that lines the mammalian Eustachian tube**. H&E stain of a longitudinal section of a chinchilla Eustachian tube with inset images of the proximal (nasopharyngeal) and distal (tympanic) portions, demonstrating the relative distribution of expression of cBD‐1 and TLR‐4 in this anatomical niche. Labeling of cBD‐1 (green color in the upper row of inset images) is greater near the colonized nasopharyngeal orifice of the Eustachian tube than at the portion closest to the middle ear, which is generally considered to be a sterile site. Conversely, labeling of TLR‐4 (green color in the lower row of inset images) is greater at the distal versus the proximal portion of the Eustachian tube.

Of interest, we observed recently that there is a marked gradient of expression of cBD‐1, an important effector of innate immunity in the uppermost airway, wherein expression is greatest at the *proximal* end of the Eustachian tube, a site of heavy colonization by NP flora (**Fig. 1**, upper row of images). Conversely, the gradient for expression of TLR‐4, a host cell‐expressed pattern recognition receptor involved in multiple functions, including detection of bacterial endotoxin [123], is the opposite (Fig. 1, lower row of images). TLR‐4 expression is thus greatest at the *distal* end of this tubal organ, wherein this anatomic site is normally considered to be sterile. In keeping with the hypothesis put forth by Munford and Varley [124, 125], this observation could well be explained by the fact that the mammalian host has evolved in partnership with its normal flora so as not to detect the presence of said microbes as a “danger” to the airway during benign colonization. Heavier expression of a defensin at the proximal end of the Eustachian tube could thus serve to maintain the bacterial load at a colonizing, as opposed to infectious, level, thereby contributing to the diverse mechanisms by which the Eustachian tube defends the middle ear.

However, during compromising conditions, such as a concurrent URT viral infection, during which it is known that members of the normal NP flora increase in concentration and also gain access to privileged sites (such as the middle ear), the host is also prepared to detect the presence of these microbes as such sites and provide the needed danger signals. This event could be triggered by the presence of bacteria at the distal end of the Eustachian tube via the demonstrated heavier expression of TLR‐4.

RSV and perhaps other URT virus‐mediated dysregulation of expression of effectors of innate immunity at the proximal or nasopharyngeal orifice of the Eustachian tube could thus contribute to weakening the effectiveness of this primary defense organ of the middle ear, thereby promoting retrograde movement of bacteria resident within the NP into the middle‐ear space wherein OM is induced.

## FUTURE AREAS OF OM RESEARCH

Remaining gaps in our understanding of the complex microbe‐host inter‐relationship that underlies the pathogenesis and resolution of OM have hampered our forward progress in terms of ability to devise novel methods to treat and preferably prevent OM. To overcome this bottleneck, there are ongoing efforts worldwide to better understand the microbes involved, with a particular focus on the molecular mechanisms of pathogenesis. Transcriptional profiling studies to decipher which virulence determinants are being expressed by the pathogens involved and in response to which microenvironmental cues, as well as how the host is responding in kind, are a robust area of investigation in the OM research community. Investigation into the role of the more recently identified human metapneumovirus and human bocavirus [72] in OM is another ongoing research focus, as are attempts to define the immune correlates of protection, operational in the pediatric population at risk for OM. Efforts to dissect the microbiome and proteome of the human URT and its secretions are expected to be robust in the coming years as well. Similar efforts to better understand the healthy and diseased oral cavity via improved knowledge of the microbes involved as well as effectors of innate and acquired immunity operational in that anatomic niche [126, 127, 128] have led to tremendous forward progress in this regard and could be expected to be equally fruitful in OM research.

## FUTURE DIRECTIONS FOR TREATMENT AND PREVENTION OF OM

Many groups are similarly involved in the development and testing of novel methods to treat or prevent OM. In terms of novel treatments, there are likely to be investigations into the potential to use small molecule inhibitors to better control the microbes involved in OM as well as to interfere in quorum‐sensing pathways that contribute to bacterial biofilm formation. The largely accepted role of biofilms in the pathogenesis of OM has led to research into how one might prevent, eradicate, or otherwise disrupt bacterial biofilms resident within the middle ear and/or present on the surface of tympanostomy tubes as a novel therapeutic approach for OM. In terms of prevention, vaccine development efforts for the viral and bacterial copathogens of OM remain a highly active area of investigation worldwide [129]. In addition to traditional (parenteral) vaccination approaches, several groups are invested heavily in assessing noninvasive methods of immunization for the prevention of OM (i.e., IN, oral, transcutaneous, sublingual), as well as better ways to adjuvant these formulations to induce an optimal immune response in a neonate, which presents multiple challenges innate to the immunological immaturity of an infant and as a result of competing maternal antibodies.

## CONCLUDING REMARKS

It is well established that URT viruses play a significant role in the development of bacterial OM, thereby making this highly prevalent pediatric disease truly polymicrobial. The mechanisms that underlie the synergistic relationship between URT viruses and bacteria in the OM disease course are not yet understood completely but in general, are predicated on overall impairment of primary host airway defenses. This complex multifactorial and polymicrobial disease presents many challenges to our ability to understand pathogenesis and immune‐mediated resolution at the molecular level as well as to devise better methods to treat or prevent the full spectrum of diseases known as OM. These challenges have led to multiple novel areas of investigation that promise to be highly informative in the coming years.

## AUTHORSHIP

This review article was written by Dr. Lauren O. Bakaletz.
